# Effect of *Haemophilus influenzae* Type b Vaccination on Nasopharyngeal Carriage Rate in Children, Tehran, 2019

**DOI:** 10.1155/2021/4923852

**Published:** 2021-03-15

**Authors:** Sedigheh Rafiei Tabatabaei, Sara Mohammadzadeh, Seyed Mohsen Zahraei, Sussan Mahmoudi, Ghazaleh Ghandchi, Seyedeh Mahsan Hoseini-Alfatemi, Abdollah Karimi, Ahmadreza Shamshiri

**Affiliations:** ^1^Pediatric Infections Research Center, Research Institute for Children's Health, Shahid Beheshti University of Medical Sciences, Tehran, Iran; ^2^Center for Communicable Disease Control, Ministry of Health and Medical Education, Tehran, Iran; ^3^Department of Epidemiology and Biostatistics, School of Public Health, Tehran University of Medical Sciences, Tehran, Iran

## Abstract

**Background:**

*Haemophilus influenzae* (*H. influenzae*) strains, which commonly reside as commensals within the human pharynx and can remain as an asymptomatic carrier, but become invasive leading to pneumonia, septic arthritis, or meningitis. The Pentavac (pentavalent vaccine, manufactured by India, SII (DTwP-HepB-Hib)) was introduced to the Iranian National Immunization Plan in November 2014. The aim of this study is to investigate *H. influenzae* type b (*Hib*) carrier rate among children under 6 years old in Tehran.

**Methods:**

This cross-sectional study was performed on 902 children including vaccinated/unvaccinated in the age of 6 months to 6 years, in Tehran. Sampling was performed from July 2019 to September 2019. Nasopharyngeal samples were taken from children by sterile swab. The PCR method was used to extract DNA. Then, all *H. influenzae* isolates were initially confirmed by molecular tests. *BexA* was used to distinguish typeable *H. influenzae* strains from nontypeable *Haemophilus influenzae* (*NTHi*).

**Results:**

A total of 902 children were enrolled in the study: 452 were female (51%). *H. influenzae* carriage rate was 267 (29%), of that 150 samples (16.6%) were typeable. The nasopharyngeal *Hib* carrier rate in the children was 2.6% (24/902). 262 cases did not receive *Hib* vaccine. Analysis in nonnursery's children aged 4 to 6 (unvaccinated) years showed that the lower educational level of father, mother, and family number correlated with increased odds of colonization of children with *Hib*.

**Conclusion:**

Our findings showed a significant decrease (60%) in the overall *Hib* nasopharyngeal carriage in healthy children under six years after 5 years after the start of Hib vaccination.

## 1. Introduction

*H. influenza*e is a Gram-negative Coccobacillus that is commonly found as part of the natural flora of the upper respiratory tract in humans, especially in healthy children [1]. These bacteria can be colonized in the throat of healthy children as an asymptomatic carrier and persist for several months, and intravascular invasion colonization of this bacterium can cause severe invasive diseases such as meningitis, septic arthritis, epiglottitis, purulent pericarditis, and pneumonia, especially in children under 5 years [1–3].

According to the type of capsular polysaccharides, these bacteria are classified to six serotypes called a, b, c, d, e, and f, identified by agglutination in the presence of a specific serum for each serotype [4], and one type without capsule or nontypeable *H. influenzae* (*NTHi*) that cannot be serotyped by formal agglutination with a specific antiserum [5].

The nasopharyngeal carries of the organism play an important role in the transmission of *Hib*, the pathogenesis of *Hib* diseases, and the emergence of immunity against bacteria. Then, this is colonized for a long time and caused respiratory tract infection, or entered the bloodstream when passing through the mucosa, and caused invasive diseases [1, 2].

According to the World Health Organization (WHO) report, in 2000, one of the two main causes of preventable diseases in children under 5 was *Hib* [2]. Carrier rate of *H. influenzae* in vaccinated children of Nepal reported 5% [6].

*Hib* vaccination coverage increased in recent years. Since 2013, about 98% of the WHO member states and 52% of infants, globally, received *Hib* vaccine by immunization programs [7].

The Hib Vaccination decreases the colonization of the Hib, and several studies have shown that other serotypes increase [2]. Currently, protein-polysaccharide conjugate *Hib* vaccines have been incorporated into the routine immunization program in almost all countries around the world [8]. Hib carrier rate in healthy children under five years in Brazil was rare after 10 years of the Hib vaccine [9].

Studies in different countries have shown that introduction of the *Hib* vaccination has reduced *Hib* carrier, in both developed and developing countries [3, 8, 10–16]. The Pentavac (pentavalent vaccine, manufactured by India, SII) entered the Iranian National Immunization Plan, in November 2014, that includes diphtheria, tetanus, whole cell pertussis, hepatitis B, and *H. influenza type b* vaccines administered three times at months 2, 4, and 6 of infants' age [17].

Therefore, the evaluation of nasopharyngeal colonization of vaccinated children can help us to determine the extent of the effect of *Hib* vaccine on this colonization compared to the similar study before vaccination. In this study, we aimed to investigate the prevalence of Hib carrier in healthy children under 6 years of age.

## 2. Subjects, Materials, and Methods

The present cross-sectional study was conducted in four health centers of northern Tehran (Ershad, Sheibani, Torab, and Samarghandi). Sampling was performed on vaccinated >(Pentavac vaccine, India, SII (DTwP-HepB-Hib)) and unvaccinated children 6 months to 6 years of age. Sampling was carried out continuously, in health centers in northern Tehran from July to September 2019. Children's information is collected by using a questionnaire. Parental oral consent was obtained. None of the participants included in our study had any type of illness on the date of specimen collection and none of them had received antibiotic therapy in preceding 4 weeks. Children who were ill at the time of the study were excluded. Nasopharyngeal samples were collected with sterile cotton swabs and immediately dropped in TSB medium then referred to the laboratory of the Pediatric Infections Research Center of the Mofid Children's Hospital.

The study protocol was approved by Pediatric Infections Research Center (PIRC) of Shahid Beheshti University of Medical Sciences. The ethical approval code is No. IR.SBMU.MSP.REC.1395.551.

### 2.1. PCR and Molecular Identification

The molecular identification of *H. Influenzae* samples was confirmed by using PCR and amplification of the *omp6* gene by specific gene primers. First, the DNA extraction method of bacterial nucleic acids was based on the DNA Kit protocol (Sinaclon, Iran) and DNA extraction product was stored in a -70°C freezer. *H. Influenzae* ATCC 49766 was used as positive control for *Hib.* Finally, PCR was used for serotyping and determination of *Hib*. PCR procedure was performed as follows: an initial denaturation at 95°C for 5 min, 40 cycles of denaturation at 95°C for 30 s, annealing at 58°C for 30 s, elongation at 72°C for 45 s, and a final extension step at 72°C for 7 min. The resulting PCR products were stained with Red safe (cat no: 1005.50) for 20 min. Then, specific bands were visualized on gel electrophoresis of PCR products under UV illumination.

To confirm species identification, PCR amplification was performed to detect the omp6 gene from genomic DNA [18]. Additionally, the PCR amplification was further carried out using the primer pair specific to *bexA* (a fragment of 354 bp in length) genes to distinguish encapsulated *H. influenzae* strains from *NTHi* strains. To determine the capsular genotypes, or phylogenetic relationships, of the *Hib* strains, PCR amplification was performed with two sets of primers designed by Ueyama et al. [18].

### 2.2. Statistical Analysis

Categorical data were reported as raw and relative abundance and quantitative data assuming normal distribution as mean and standard deviation. Simple and multiple logistic regression was used to analyze factors associated with colonization frequency with Hib (two-state qualitative variable). Given that the day-nursery variable was identified as an effect modifier, data analysis was performed separately for nursery's children and nonnursery's children. Statistical significance was considered less than 0.05.

## 3. Results

### 3.1. Descriptive Data

The demographic and baseline values of samples are presented in Table 1. A total of 902 children were enrolled in the study: 452 were female (51%) and 450 were male.

146 sample of children under 1 year, 154 samples under 1-2 years, 153 samples under 2-3 years, 150 samples under 3-4 years, 151 samples under 4-5 years, and 148 samples under 5-6 years were taken. Given that the vaccine program has been started in 2014 in Iran, children over the age of 4 and some children in other age groups did not receive the vaccine. So, because of the starting time of the vaccination and some other reasons, 262 cases have not received Hib vaccine, which includes 6 children (one year old), 5 children (two years old), 16 children (three years old), 34 children (four years old), 56 children (five years old), and 145 children (six years old).

In PCR assays, 267 (29%) of the 902 samples were *H. influenzae*, of that 150 (56.17%) encapsulated and type-able isolates have been reported. Among the 640 children who received the vaccine and among the 262 who did not receive vaccine, 15 (2.3%) and 9 (3.4%), respectively, were reported as *Hib*.

### 3.2. Factors Associated with Colonization of *Haemophilus influenzae Type b*

The mean age in colonized children was 4.25 ± 1.29 and in non-colonized children was 3.48 ± 1.70. As shown in the table, in the data analysis (multivariable), the only variable that has a significant association with colonization of *Hib* is the level of maternal education.

### 3.3. Data Analysis in Nursery's Children

Data on nursery's children are presented in Table 2. As shown in this table, none of the variables studied in preschool children had a significant relationship with the colonization of *Hib*.

### 3.4. Analysis in Nonnursery's Children

Multivariable analysis showed that aged 4 to 6 years (compared to age 1 to 3 years) (adjusted OR = 4.25; 95% CI: 1.11-16.33; *p* value = 0.04), lower educational level of father (adjusted OR = 3.72; 95% CI: 0.97-14.32; *p* value = 0.056), and family number (adjusted OR = 3.72; 95% CI: 0.97-14.32; *p* value = 0.056) correlated with increased odds of colonization of nonnursery's children with *Hib*. This analysis shows that statistically, a mother's level of education is as effective as a father's level of education (adjusted OR = 3.64; 95% CI: 0.94-14.02; *p* value = 0.061), approximately. The analysis of data in nonnursery's children is summarized in Table 3.

The day-nursery variable is an effect modifier in this study. Therefore, the data of nursery's children and nonnursery's children were analyzed separately. None of the variables studied in nursery's children had a significant relationship with the colonization of *Hib*.

## 4. Discussion

This study was conducted to investigate the prevalence of colonization of *H. influenzae* after 5-year vaccination in children under six years old, in Tehran.

The findings of this study showed that nonnursery's children aged 4 to 6 years, with low mother and father education level, and family members more than 5 are more at risk for colonization of *H. influenzae*. Carrier rate of *Hib* varies greatly from place to place.

In this study, the nasopharyngeal *Hib* carriage rate in the children was 2.6%. This is similar to reports from other developing countries in CDC reports (0.5%-3%) [7]. This prevalence is higher than the *Hib* colonization rate in some developed countries such as Canada [19], UK [13], Japan [20], and Brazil [21] after vaccination (0%–1.5%) [21]. In these countries following the introduction of routine immunization of infants with *Hib* conjugate vaccines, the carrier rate has reduced dramatically, in both vaccinated and unvaccinated children due to the herd effect [22]. On the contrary, in nasopharyngeal carriage studies using culture and identification of all *H. influenzae* serotypes before vaccination, carriage rates vary from 15% to 40% [23, 24]. It seems these findings are more than this study based on our data that no clear explanation has been provided. One study suggested that oropharyngeal culture for *Hib* carriers is more sensitive than nasopharyngeal culture. Samples were obtained from 717 healthy children younger than 6 by a single oropharyngeal swab from each participant and to streak into chocolate blood agar. The PCR was used to determine capsular genotype. The overall rate of *H. influenzae* carriage in vaccinated and unvaccinated children was 14.1%. Age, the place of study, presence of young-siblings, and complete Hib vaccination status were associated with colonization, independently [22]. In Karimi et al.'s study in Tehran in 2007 before vaccination, out of 1000 children aged ≤5 years from 25 daycare centers, the rate of *Hib* carriage by a culture method was reported as 7.6% [25]. This study approved that the vaccination has been able to reduce about 60% of the pharyngeal carrier. Karimi et al.'s result was an important estimation for determining the status of Hib colonization in Iranian children before Hib vaccination [2].

One study in Kenya showed immunization using *Hib* conjugate vaccines declined the prevalence of nasopharyngeal carriage [10]. In another study, immunization with *Hib* conjugate vaccines has also decreased the rate of colonization to less than 1% [14]. In Giufre et al.'s study in Italy in 2015, almost all *H. influenza* isolates in healthy children were *NTHi* type except 3 capsuled isolates that did not belong to vaccine-preventable serotypes. According to the results of the study, vaccination reduced or eliminated the *Hib* carriage [22]. In the current study, all children were selected from a climatic zone north of Tehran, so further investigation is needed to obtain more accurate results. In addition, in previous studies, the age and presence of young siblings were identified as independent risk factors [22]. In a study by Odutola et al. in 2013, on 1030 Gambian infants (median age 35 weeks), the little effect of age on carriage of any of the potential pathogens was observed, and with the increase of age, the prevalence of carriage with *H. influenzae* trends to increase. The finding of this study is similar to Odutola et al.'s study. Based on these findings, the mean age in colonized children (4.25 ± 1.29) was more than that in noncolonized children (3.48 ± 1.70).

In this study, none of the variables studied had a significant relationship with the colonization of *Hib*, in nursery's children. In nonnursery's children with *Hib*, age 4 to 6 years (*p* = 0.02), family number (*p* = 0.03), lower educational level of father (*p* = 0.04), and mother's level of education (*p* = 0.03) correlated with increased odds of colonization.

Although there is controversy about the correlation between the *Hib* carriage rate and other factors, such as gender, number of siblings < 5 years old, and recent respiratory disease [23]. In Puig et al.'s study, the association between gender and respiratory problems with *H. influenzae* colonization is not shown. [23]. With regard to CDC, exposure factors include household crowding, large household size, child care attendance, low socioeconomic status, low parental education levels, and school-aged sibling effect on *Hib* [7]. Similar to the CDC results, this study showed that in nonnursery's children, the rate of *Hib* colonization was related to the family number and lower educational level of father and mother. Also, in one study, in China in 2016, inverse association between household size and *Hib* carriage was shown. So *Hib* carriage was more in children with more siblings compared to single children. It indicated possible effects from human crowding. It seems crowding and having more children in the family are reasons for more Hib carriage in developing countries than in developed countries [26].

According to a study by Jalali et al. in 2014, the rate of *H. influenzae* carriers is high (28%) in children under 6 years of age in Iran and similar to unvaccinated countries. In this study, 533 mucus samples were obtained by nasopharyngeal swabs from children under 6 years old in 4 nursery centers in Tehran or refereed to the Children's Medical Center of Tehran. The *H. influenzae* diagnosis was performed by standard biochemical tests and confirmed by PCR assay. That result showed the rate of *H. influenzae* carriage was linked to age and respiratory infection diseases. The carriage rate was highest in children aged 25-48 months and decreased with increasing age [27].

This study indicated that *Hib* vaccine, as a constituent of pentavalent vaccine, successfully reduced *Hib* nasopharyngeal colonization.

One of the limitations of this study was the difficulty of swab sampling from children under two years. This can affect the outcome of the study. Moreover, in this study, the use of antibiotics was not investigated. This variable may affect the rate of *Hib* cloning.

## 5. Conclusion

Vaccination of *Hib* conjugate has been displayed to reduce the pharyngeal carriage rate of *Hib* [22]. Our results showed that compared to the carrier group, which was carried out before vaccinations in the same age group in Tehran, Iran, about 3 times (2.6 compared to 7.5), the carrier rate decreased. Thus, the vaccination has been able to reduce about 60% of the pharyngeal carriers. In unvaccinated children representing a 3.6% pharyngeal carrier, it depicts the topic of herd immunity. Following vaccinations in the target group, the carrier rate has also declined in the groups that did not receive the vaccine.

According to the 2.6% rate of *H. influenzae* after vaccination program and comparison with the rate below 1% in other countries, it was recommended that future studies investigate colonization in Iranian children.

## Figures and Tables

**Table 1:** The demographic and baseline values of samples.

Variables		*N* (%)
Age^∗^		3.50 ± 1.70
Gender	Female	452 (50.11%)
	Male	450 (49.89%)
Family number	2-3	481 (53.33)
	4	356 (39.47)
	≥5	65 (7.21)
Father education	Associate's degree, diploma, or less	296 (32.82%)
	BSc, MSc, or doctorate	606 (67.18%)
Mother education	Associate's degree, diploma, or less	284 (31.49%)
	BSc, MSc or doctorate	618 (68.51%)
Pentavalent vaccine history	No	262 (29.05%)
	Yes	640 (70.95%)
Babysitting at home or day-nursery	Home	539 (59.76%)
	Day-nursery	363 (40.24%)
Smoking (adults, past or current)	No	795 (88.14%)
	Yes	107 (11.86%)

^∗^Mean ± SD.

**Table 2:** Nursery's children data.

Variables	Groups	*H. influenzae*		Unadjusted OR	95% CI	*p* value^∗^
		Non b or negative *N* (%)	Hib *N* (%)			
Age group	1-3 yr	107 (95.54)	5 (4.46)	1	0.23-2.20	0.55
	4-6 yr	243 (96.81)	8 (3.19)	0.70		
Gender	Female	170 (94.97)	9 (5.03)	1	0.13-1.39	0.15
	Male	180 (97.83)	4 (2.17)	0.42		
Family number	2-3	225 (96.57)	8 (3.43)	1	0.36-3.51	0.84
	≥4	125 (96.15)	5 (3.85)	1.12		
Father education	Associate's degree, diploma, or less	80 (96.39)	3 (3.61)	1.01	0.27-3.77	0.99
	BSc, MSc, or doctorate	270 (96.43)	10 (3.57)	1		
Mother education	Associate's degree, diploma, or less	69 (94.52)	4 (5.48)	1.81	0.54-6.05	0.34
	BSc, MSc, or doctorate	281 (96.90)	9 (3.10)	1		
Pantavalan intake	No	125 (96.15)	5 (3.85)	1.12	0.36-3.51	0.84
	Yes	225 (96.57)	8 (3.43)	1		
History	Negative	292 (96.05)	12 (3.95)	1	0.05-3.29	0.41
	Positive	58 (98.31)	1 (1.69)	0.42		
Smoking	No	337 (96.29)	13 (3.71)	1	0.00-9.65	1.00
	Yes	13 (100.00)	0 (0.00)	1.46		
Antibiotic	No	342 (96.61)	12 (3.39)	1	0.41-30.80	0.25
	Yes	8 (88.89)	1 (11.11)	3.56		

^∗^Simple and multiple logistic regression (forward method); *p* < 0.05.

**Table 3:** Nonnursery's children data.

Variables	Groups	*H. influenzae*		Unadjusted OR	95% CI	*p* value^∗^
		Non b or negative *N* (%)	Hib *N* (%)			
Age group	1-3 yr	338 (99.12)	3 (0.88)	1	1.24-18.09	0.02
	4-6 yr	190 (95.96)	8 (4.04)	4.74		
Gender	Female	269 (98.53)	4 (1.47)	1	0.53-6.28	0.35
	Male	259 (97.37)	7 (2.63)	1.82		
Family number	2-3	246 (99.19)	2 (0.81)	1		
	4	234 (97.50)	6 (2.50)	3.15	0.63-15.78	0.16
	≥5	48 (94.12)	3 (5.88)	7.69	1.25-47.24	0.03
Father education	Associate's degree, diploma, or less	205 (96.24)	8 (3.76)	4.20	1.10-16.02	0.04
	BSc, MSc, or doctorate	323 (99.08)	3 (0.92)	1		
Mother education	Associate's degree, diploma, or less	203 (96.21)	8 (3.79)	4.27	1.12-16.28	0.03
	BSc, MSc, or doctorate	325 (99.09)	3 (0.91)	1		
Pantavalan intake	No	128 (96.97)	4 (3.03)	1.79	0.51-6.20	0.36
	Yes	400 (98.28)	7 (1.72)	1		
History	Negative	495 (97.83)	11 (2.17)	1	0.00-6.27	0.99
	Positive	33 (100.00)	0 (0.00)	0.99^∗^		
Smoking	No	436 (97.98)	9 (2.02)	1	0.22-4.95	0.95
	Yes	92 (97.87)	2 (2.13)	1.05		
Antibiotic	No	519 (97.92)	11 (2.08)	1	0.00-27.32	1.00
	Yes	9 (100.00)	0 (0.00)	3.89^∗^		

^∗^Simple and multiple logistic regression (forward method); *p* < 0.05.

## Data Availability

The required data are given in the text of the article.
